# Cell segmentation by multi-resolution analysis and maximum likelihood estimation (MAMLE)

**DOI:** 10.1186/1471-2105-14-S10-S8

**Published:** 2013-08-12

**Authors:** Sharif Chowdhury, Meenakshisundaram Kandhavelu, Olli Yli-Harja, Andre S Ribeiro

**Affiliations:** 1 Laboratory of Biosystem Dynamics, Computational Systems Biology Research Group, Department of Signal Processing, Tampere University of Technology, 33101 Tampere, Finland; 2 Institute for Systems Biology, 401 Terry Avenue North, Seattle, WA 98109-5234, USA

## Abstract

**Background:**

Cell imaging is becoming an indispensable tool for cell and molecular biology research. However, most processes studied are stochastic in nature, and require the observation of many cells and events. Ideally, extraction of information from these images ought to rely on automatic methods. Here, we propose a novel segmentation method, MAMLE, for detecting cells within dense clusters.

**Methods:**

MAMLE executes cell segmentation in two stages. The first relies on state of the art filtering technique, edge detection in multi-resolution with morphological operator and threshold decomposition for adaptive thresholding. From this result, a correction procedure is applied that exploits maximum likelihood estimate as an objective function. Also, it acquires morphological features from the initial segmentation for constructing the likelihood parameter, after which the final segmentation is obtained.

**Conclusions:**

We performed an empirical evaluation that includes sample images from different imaging modalities and diverse cell types. The new method attained very high (above 90%) cell segmentation accuracy in all cases. Finally, its accuracy was compared to several existing methods, and in all tests, MAMLE outperformed them in segmentation accuracy.

## Background

Single cell microscopy and subsequent analysis has gained much interest recently in areas ranging from studies of gene expression dynamics [1-3], to studies of cell aging [4,5] to disease classification [6]. However, as most processes in cells are stochastic in nature [7] their study requires high-throughput measurements and analysis. The manual extraction of the results from the raw image data is thus prohibitive, causing a need for accurate and robust methods of cell segmentation.

Most existing methods lack in generic applicability and require strong assumptions on cell features i.e. cell shape, size, etc. Additionally, their performance is highly sensitive to cell density and signal to noise ratio. One of the presently most successful and used cell image analysis tools is 'Cellprofiler' [8], an open source software platform for automated cell segmentation from microscopy images. Cell segmentation in Cellprofiler is performed in two steps. First, it separates objects from image background by thresholding. Next, the clumped objects are segmented again by considering intensity or shape as a feature for discrimination. Cellprofiler provides several alternatives for automated threshold selection and clumped cell segmentation. The major drawback of its segmentation algorithm is that its accuracy decreases significantly when cells are in large, dense clumps.

Another state of the art software tool is 'Schnitzcells' [9]. Schnitzcells provides solutions for segmentation and tracking of *Escherichia coli *cells from images by confocal or phase contrast microscopy. The segmentation of cells in Schnitzcells is a multi-stepped process. First, it applies edge detection for generating initial segmentation. Next, it splits long or clumped cells. Finally, it considers too small objects as false positives and discards them. The major problem is the large number of parameters that, without proper tuning, cause the accuracy of the segmentation to decrease notably. Further, it has a limited scope of application, i.e. it only handles *E. coli *and *Bacillus subtilis *cells and often presents a significant number of false positives.

Finally, it is worth mentioning the cell segmentation algorithm for histopathology images, whose implementation was made available in the Farsight toolkit [10]. This method exploits graph-cuts-based segmentation for segmenting foreground signals from the image background. Then, the nuclear seed points are detected by a multi-resolution edge detection method. Aside these, other methods for cell segmentation were proposed (see e.g. [11,12]). In general, these split the overall segmentation task into three steps. First, a separation of foreground objects from image background is made. Next, a post processing step is applied to split the under-segmented clumped cells. Finally, false positives are discarded by some criteria.

Here, we propose a novel cell segmentation method, MAMLE, which maintains very high cell segmentation accuracy in dense cell clusters with low signal to noise ratio (SNR). Moreover, MAMLE requires very few assumptions on cell shape or size, thus, it can handle a wide range of cell types in different imaging modalities. MAMLE is novel in that i) it adopts a state of the art image denoising technique for improving SNR in image, ii) unifies multi-resolution edge detection and threshold decomposition to accomplish the initial segmentation iii) corrects the over-segmented and under-segmented cells based on likelihood estimate, which is shown to be adaptive to varying conditions. Above all, MAMLE innovates in that it learns different shape features on the fly and exploits the learnt parameters for cell segmentation correction. A properly combined usage of all features is implemented to obtain robust and accurate cell segmentation.

MAMLE is primarily targeted towards one of the most challenging cell types for automated segmentation, *E. coli*, a model organism in cell and molecular biology research [13-15]. The high division rate, the formation of dense colonies and the cells' morphology make the segmentation more challenging than for most other cell types. We first describe the method, after which we evaluate its cell segmentation accuracy and compare it with state of the art cell segmentation platforms. Next, the robustness of MAMLE is studied in its parametric space. In the end, we present our conclusions.

## Methods

**Figure 1:**
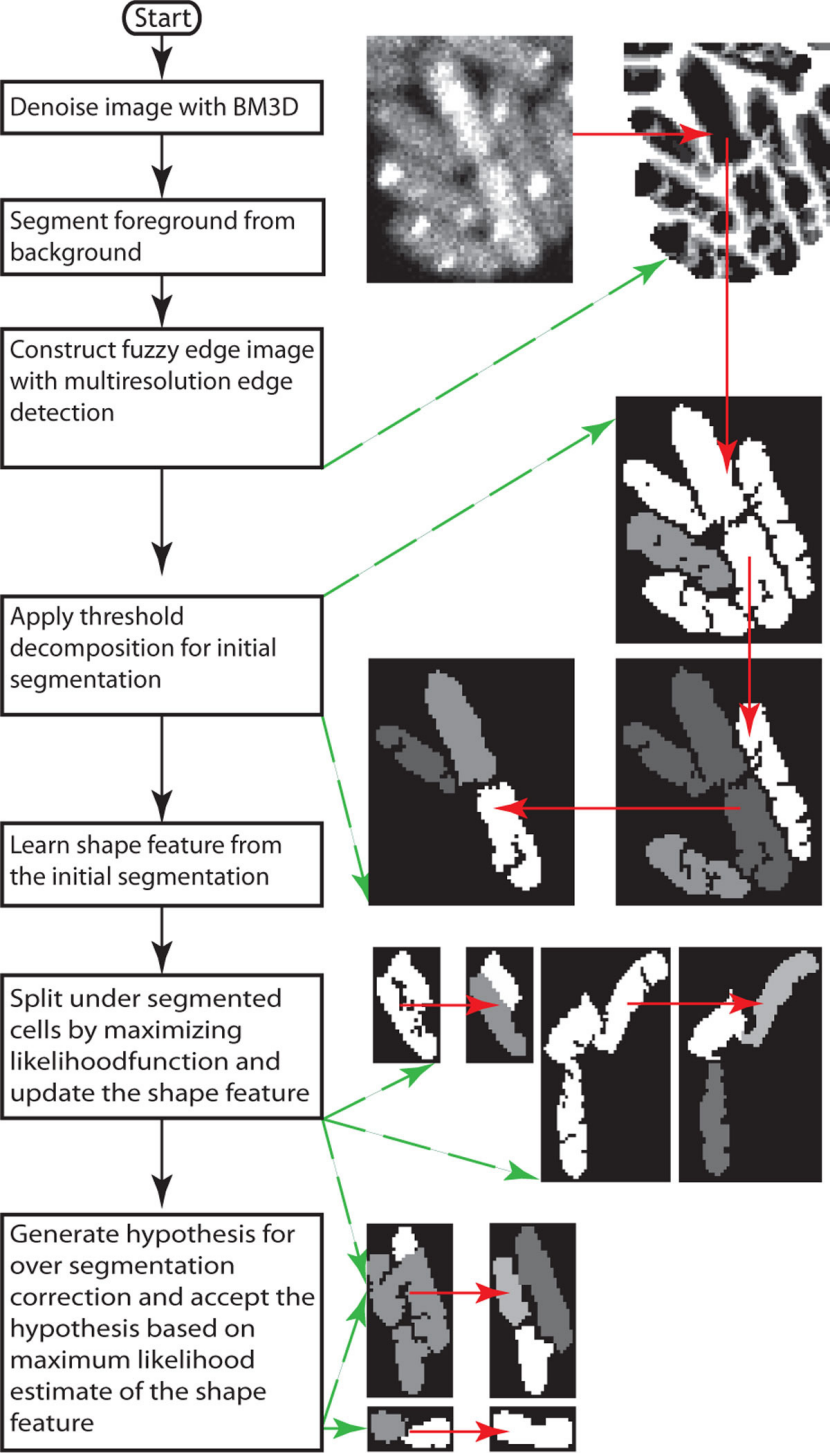
**Schematic flow chart of the proposed method**. The green arrow points to the result of the respective operation and the red arrow indicates the input/output data flow from one operation to another.

MAMLE cell segmentation method comprises 7 steps: i) image denoising, ii) foreground and background segmentation, iii) multi-scale morphological edge detection, iv) threshold decomposition and initial segmentation, v) shape learning form the initial segmentation, vi) likelihood optimization based splitting and vii) maximum likelihood based merging. A flow chart of the algorithm is illustrated in Figure 1. Next, we describe each step in detail:

i) **Image denoising: **fluorescent images often have low SNR, which leads to cell detection artifacts. Hence, denoising filters are often applied as a pre-processing step for segmentation. MAMLE exploits a state of the art image denoising technique known as Block-Matching and 3D filtering (BM3D) [16]. We opted for BM3D due to its balance between noise cancellation and edge preservation capability [16]. BM3D splits a noisy 2D image into fixed size blocks (8x8) and searches for the blocks that match the reference block. The matching blocks are arranged into a 3D array, known as 'group'. On each group, a 3D transformation is executed and thresholded. Afterwards, BM3D inverse transforms the group and aggregates it with weights to augment the basic estimate. The augmented basic estimate acts as a pilot signal for subsequent steps. Finally, a collaborative Wiener filtering is executed on the noisy signal to obtain the noise removed image.

ii) **Foreground and background segmentation: **this step (second step in Figure 1) separates individual cell colonies from the image background. In fluorescence microscopy, cells are stained with fluorophores or they express a fluorescent protein. As a result, the image background appears darker than foreground objects (i.e. cells). Therefore, a block-wise Otsu threshold, followed by bilinear interpolation, is applied to separate each cell colony from the background [8,17]. Phase contrast images, on the other hand, have different intensity profiles for foreground and background. For these, we use iterative range filtering to segment foreground objects from the image's background [18]. In both cases, the extracted foreground mask is used to select the region of interest.

iii) **Multi-scale morphological edge detection: **this is a key step of MAMLE (third step in Figure 1). Recent studies showed that multi-scale edge detection has, in general, a more robust performance than single scale edge detection strategies [10], particularly when combined with morphological operators [18,19]. We propose a novel multi-scale morphological edge detector to generate the fuzzy edge image for cell segmentation. An edge in a digital image ( f) can be realized as a local intensity minimum with direction. Thus, a pixel at an edge should not be altered by the morphological erosion operation. With this assumption, a binary edge ($E_{s}^{}\left(f\right)$) image at scale level *S *can be defined as in (1) and (2).

(1) $$E_{s}\left(f\right)=\underset{d}{⋃}E_{s}^{d}\left(f\right)$$

(2) $$E_{s}^{d}(f)={\{}_{0}^{1\;if\;f=f\text{o}{\text{B}}_{\text{s}}^{\text{d}}}$$

where $E_{s}^{d}\left(f\right)$is the edge image in the direction d  and the symbol (∘ ) represents the morphological erosion operator with the respective support ${\text{B}}_{s}^{d}$. The direction *d *is such that that it allows choosing four possible directions, 1 to 4, corresponding to horizontal, vertical, diagonal from left to right, and diagonal from right to left, respectively. The support ${\text{B}}_{s}^{d}$, at scale level s and in direction d , can be defined as in (3) - (6).

(3) Bs1=[1 01×s-11 01×s-11]

(4) $${\text{B}}_{s}^{2}={\left({\text{B}}_{\text{s}}^{1}\right)}^{\text{T}}$$

(5) $${\text{B}}_{\text{s}}^{3}{=\text{B}}_{\text{s}}^{1}{\text{I}}_{2\text{s}+1\times 2\text{s}+1}$$

(6) $${\text{B}}_{\text{s}}^{4}=R_{\frac{\pi }{2}}\left({\text{B}}_{\text{s}}^{3}\right)$$

Here, ${\text{0}}_{1\times s-1}$ is a row vector of zeros of the size s-1, ${\text{I}}_{2s+1\times 2s+1}$is the identity matrix of the size 2s+1, T is the transpose operator and $R_{\frac{\pi }{2}}\left(\left|.\right|\right)$ is the rotational operation. The fuzzy edge image is computed as (7)

(7) $$E\left(f\right)=\overset{S}{\underset{s=1}{\sum }}E_{s}\left(f\right)$$

Each pixel in the fuzzy edge image (E(f)) is a real valued integer within the range 0 to S, where S is the maximum scaling factor. A pixel on the most certain edges in the original gray scale images holds a value close to S, while a pixel on the smooth region holds a value close to zero in the fuzzy edge image.

iv) **Threshold decomposition: **the obtained fuzzy edge image is treated as an initial estimate of intensity edge in the grayscale image (forth step in Figure 1). Like more traditional edge detection algorithms [20], MAMLE thresholds this fuzzy edge image to obtain edges for cell segmentation. However, even with an exhaustive search, we were unable to obtain a rational threshold value for selecting edges from the fuzzy edge image. Therefore, we use instead an adaptive method for threshold selection, namely, a 'threshold decomposition' technique [21], which increases or decreases the threshold by a constant amount within an interval for a fixed number of times. Mathematically, this procedure can be expressed as (8) [21].

(8) $$T^{i}\left(f\right)=\left\{\begin{array}{c} 1\;if\;f\geq i\\ 0\;if\;f<i \end{array}\right)$$

The decomposition starts with the strongest threshold (i=S) that subdivides the foreground object, based on most certain or strongest edges. Afterwards, it lowers the threshold gradually to a predefined bound, unless the foreground object is already reduced to a size smaller than a predefined value (i.e. the expected maximum size of a cell). The threshold decomposition is recursive and the exact number of decomposition levels is specific for each of the detected objects. The details are listed in 'Algorithm 1'. The segmentation result is treated as an initial segmentation mask for the following steps. As noted, the initial segmentation results show several over and under-segmentation artifacts, which should be corrected.

**Algorithm 1**: ThresholdDecompositon(E,f)

Input: fuzzy edge image(E ), edge threshold(f ), area threshold $T_{a}$

1. Initialize: S←∅

2. Select the edges that are stronger than f  and label the image based on edge selection

3. ASSIGN $f^{-}\leftarrow f-1$

4. FOR EACH labelled region ${\text{S}}_{i}$

 a) IF area (${\text{S}}_{i}$) >$T_{a}$ THEN

 a.1) UPDATE S←S∪ ThresholdDecompositon($\text{E}\cap {\text{S}}_{i},f^{-}$);

 b) ELSE

 b.1) UPDATE $\text{S}\leftarrow \text{S}\cup {\text{S}}_{i}$

5. Return S ;

v) **Learning shape parameter: **First, we need to acquire a morphological or shape feature from the initial segmentation. As the initial results are partially correct, they can be used to obtain different shape features. Afterwards, they can be categorized into classes, namely 'correct segmentation', 'under-segmentation' or 'over-segmentation', based on the morphological features. However, to maintain the classifier tractable, we treat each object as a simple polygon and consider its area, the major axis length and the minor axis length as the discriminant features for classification. The major (a) and the minor (b) axes are computed according to (9) and (10) [22].

(9) $$a=4\sqrt{\frac{m_{00}m_{11}-m_{01}m_{01}}{A\lambda_{1}}}$$

(10) $$b=4\sqrt{\frac{m_{00}m_{11}-m_{01}m_{01}}{A\lambda_{2}}}$$

Here, $m_{xy}$ is the centroidal moment, A  is the area and $\lambda_{1}$, $\lambda_{2}$ are two orthogonal eigenvalues of the polygon. The ideal cell shape and shape distribution are assumed as multivariate Gaussian distributions on the feature space. The parameter of this distribution can be estimated from the sample mean vector and the covariance matrix in the feature space of the initial segmentation results.

vi) **Binary split: **this step (sixth step in Figure 1) is the first that re-evaluates the initial segmentation results for correction. Binary split considers the initially segmented cells that are larger than the average cell size as a candidate for the split. The splitting is done by maximizing the likelihood function. The log-likelihood of a detected object to be a cell is formulated as (11).

(11) $$\begin{array}{cc} ll\left({\text{X}}_{\text{i}}\right) & =\text{log}\left(\frac{1}{{\left(2\pi \right)}^{\frac{d}{2}}{\left|\text{Σ}\right|}^{\frac{1}{2}}}e^{-{\left({\text{X}}_{\text{i}}-\mu \right)}^{\text{T}}{\text{Σ}}^{-1}\left({\text{X}}_{\text{i}}-\mu \right)}\right)\\ & =\text{log}\left(\frac{1}{{\left(2\pi \right)}^{\frac{d}{2}}{\left|\text{Σ}\right|}^{\frac{1}{2}}}\right)-{\left({\text{X}}_{\text{i}}-\mu \right)}^{\text{T}}{\text{Σ}}^{-1}\left({\text{X}}_{\text{i}}-\mu \right) \end{array}$$

Here, μ and Σ are the mean and covariance of the multivariate Gaussian distribution, estimated from the initial segmentation. Since the covariance matrix is invariant with respect to the object under selection, the log-likelihood function can be simplified as (12).

(12) $$D\left({\text{X}}_{\text{i}}\right)={\left({\text{X}}_{\text{i}}-\mu \right)}^{\text{T}}{\text{Σ}}^{-1}\left({\text{X}}_{\text{i}}-\mu \right)$$

A closer look at the objective function ($D\left({\text{X}}_{i}\right)$) reveals that, given the considered metric, the minimization of the variance of the normalized distance from the mean vector (μ ) would maximize the likelihood function. This problem is usually realized as a Gaussian mixture model problem and is solved with the expectation maximization (EM) algorithm [23]. However, we did not consider EM as a solution since: i) EM needs to know the number of existing mixtures, ii) EM does not have direct control on the shape of the distribution and, iii) since EM considers global optimization, there is no straight forward way to consider the case where a part of one cell is clumped with one or more cells. Thus, we formulate an iterative procedure for likelihood maximization that splits a clumped object into two parts by maximizing the likelihood only in one of the parts, disregarding the other.

After the split, the disregarded part is reconsidered for split and processed recursively, unless its size is already smaller than the average cell size. The split procedure is not completely flawless. Occasionally, it over-segments a single cell into multiple parts. Nevertheless, most over-segmented cells are re-merged in the subsequent merge procedure.

vii) **Over-segmentation correction: **this step (seventh and eight steps in Figure 1) merges the over-segmented cells based on a maximum likelihood estimate of the shape feature vector. The maximum likelihood estimate based merging is obtained by transforming the problem into a binary integer programming problem [24]. Similar approaches have been used for cell tracking [25]. The merging scheme first constructs a candidate set (C ) for merging. Each member in the candidate set needs to satisfy the logical quantifier expressed in (13).

(13) $$\forall c_{i}\in C\exists c_{j}\in C\left(\begin{array}{c} \;\;\;\;D\left({\text{X}}_{i}\right)+D\left({\text{X}}_{j}\right)\geq \\ D\left({\text{X}}_{ij}\right)\wedge c_{i}\neq c_{j} \end{array}\right)$$

Here, $c_{i}$is a cell or part of cell identified in the prior steps and ${\text{X}}_{i}$ is the respective feature vector. ${\text{X}}_{ij}$ represents the feature vector of the object $c_{i}$ merged with $c_{j}$. The first $\left|C\right|$ rows of the hypothesis matrix H  and likelihood vector L  are initialized as (14) and (15) respectively.

(14) $$\text{H}\left(i,j\right)=\left\{\begin{array}{c} 1\;if\;i=j\\ 0\;if\;i\neq j \end{array}\right)$$

(15) $$\text{L}\left(i\right)=-D\left({\text{X}}_{i}\right)$$

Subsequently, it generates all possible hypotheses of merging two objects from the candidate list by satisfying the merging constraint (16). Additionally, for each of the accepted hypotheses, a single row is appended in the hypothesis matrix according to (17) and an element in the likelihood vector is added as (18).

(16) $$\forall c_{ij}\in C_{2}\exists c_{i},c_{j}\in C\left(D\left({\text{X}}_{i}\right)+D\left({\text{X}}_{j}\right)\geq D\left({\text{X}}_{ij}\right)\wedge c_{i}\neq c_{j}\right)$$

(17) $$\text{H}\left(h^{\prime },k\right)=\left\{\begin{array}{c} 1\;if\;\;\;\;\left(k=i\vee k=j\right)\wedge c_{ij}\in C_{2}\\ 0\;other\;wise \end{array}\right)$$

(18) $$\text{L}\left(h^{\prime }\right)=-D\left({\text{X}}_{ij}\right)$$

Similarly, it generates hypothesis list ($C_{3}$) for merging three objects and adds a single row in the hypothesis matrix and likelihood vector for each of the generated hypotheses. This can be extended further, beyond the third level. We did not find any evidence to justify such expansion. Hence, we limited the hypothesis generation process to level three. The generated hypothesis matrix has $m=\left|C\right|+\left|C_{2}\right|+\left|C_{3}\right|$ rows and $n=\left|C\right|$ columns, while the likelihood vector has m  rows. Given this, the maximum likelihood estimate for merging can be obtained by selecting the hypotheses that includes each of the identified objects exactly in one hypothesis and, at the same time, maximizes the total likelihood. This problem can be solved by solving a standard binary integer programming problem formulated in (19).

${\text{x}}^{*}=\text{arg }\underset{\text{x}}{\text{max}}\left({\text{L}}^{\text{T}}\text{x}\right)$ such that ${\text{H}}^{\text{T}}{\text{x}}^{*}=\text{1}$ (19)

Here, ${\text{x}}^{*}$ is a binary column vector that indicates whether the respective hypothesis should be accepted or not and 1 is a column vector of ones that restricts the inclusion of each candidate exactly in one hypothesis. However, the stated problem belongs to the class of NP-hard problems [24]. Thus, there is no known polynomial time solution for solving it. We exploited a linear programming (LP)-based branch-and-bound technique to obtain an approximate solution [24]. Finally, the selected hypotheses are accepted and the objects are merged to construct the final segmentation result.

## Materials

*E. coli *DH5α-PRO strain containing a bacterial expression vector PROTET-K133 carrying the MS2-dimer (MS2d) fused with green fluorescent protein (MS2d-GFP) was used for this study [26,27]. This vector has an inducible promoter P(LtetO-1), which is under tight regulation of anhydrotetrachycline (aTc, IBA GmbH, Göttingen, Germany). Constructs were generously provided by Dr. Ido Golding, University of Illinois. Cells were grown in LB medium, supplemented with kanamycin antibiotic for the selection of cell containing the P_LtetO-1_-Ms2d-GFP plasmid. For full induction of gene expression, cells were grown overnight at 37 °C with aeration to reach an optical density of OD600 ≈ 0.3-0.5. The cells were incubated with inducer aTc (100 ng/ml) for 45 minutes to attain full induction of MS2d-GFP. Following induction, a few micro litres of culture were placed between a cover-slip and a thick slab of 1% agarose containing LB. Microscopy was performed at room temperature (22 °C) using a Nikon Eclipse (TE-2000-U, Nikon, Tokyo, Japan) inverted confocal laser-scanning microscope equipped with a 100X magnification (1.5NA) objective. GFP fluorescence was measured using a 488 nm laser (Radius 405 laser, Coherent, Inc., Santa Clara, CA) and a 515/30 nm detection filter (100-120 detector gain).

In case of *Staphylococcus aureus*, cells were grown in LB medium. OD600 ≈ 0.3-0.5 cells were incubated with 0.5 mg ml-1 DNA binding stain, 4'6-diamidino-2-phenylindole (DAPI, Sigma) for 1 hour at 37 °C and centrifuged at 7000 RPM for 10 min. Cell pellet was diluted 1:100 time and few micro litres of cells were placed in a microscopic slide as mentioned above to perform the image acquisition. DAPI stain expression was measured using a 406 nm laser (Radius 405 laser, Coherent, Inc., Santa Clara, CA) and a 450/35 nm detection filter (100-120 detector gain).

## Results

**Figure 2:**
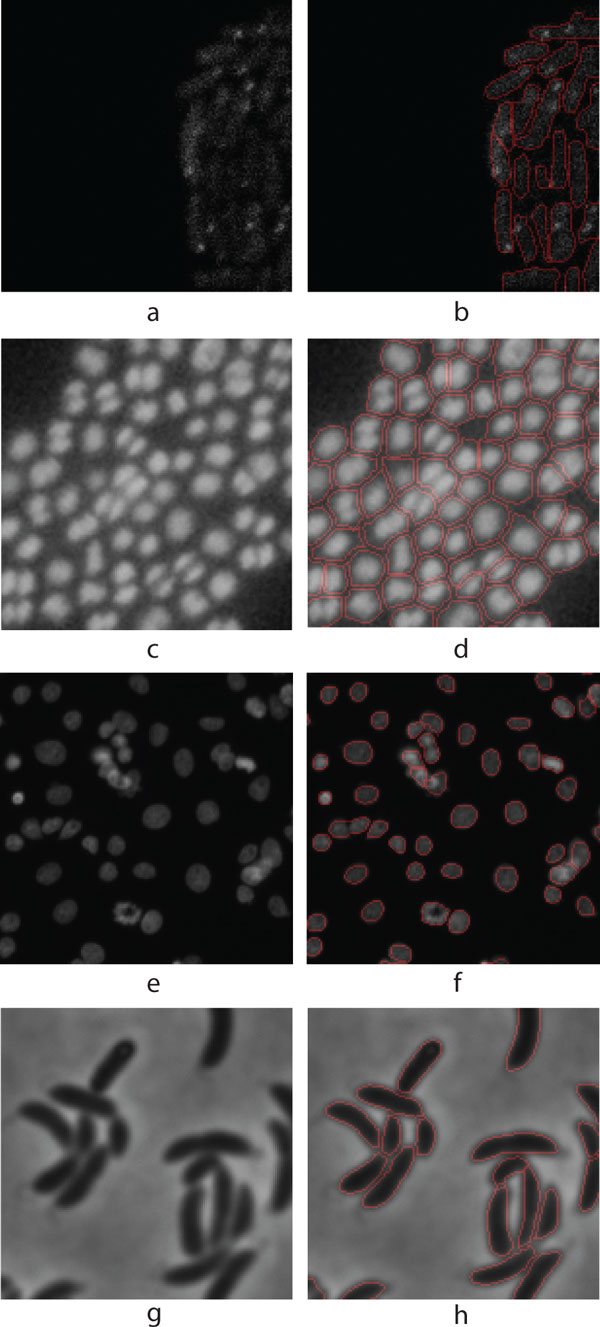
**Illustrative examples from the test samples**. (a) Fluorescent protein labelled *E. coli *cells captured with confocal microscope, (b) Segmented result of (a), (c) Fluorescent protein labelled *Staphylococcus *cells in Epifluorescence microscopy image. (d) Segmented result of (c), (e) Human HT29 Colon Cancer 1 image set (Source [8]), (f) Segmented result of (e), (g) *E. coli *cells captured with phase contrast microscope (Source [11]), (h) Segmented result of (g).

We carried out an empirical evaluation of the algorithm with several test sets. The results are categorized into four classes: i) true positive (TP), if a cell is segmented properly; ii) over-segmentation, if a cell is split into more than one piece; iii) under-segmentation, if more than one cell is recognized as a single cell; and iv) false negative (FN), if a clearly visible cell is not detected. Apart from these, some cells were undergoing division which, depending on the stage, is classified as a single cell or as two independent cells, according to the specifications of the algorithm (these classifications are not considered in errors estimation). A small fraction of detections were false positives (less than 0.1%), and since the overall contribution of false positives is insignificant, we discard this result from the evaluation. The results from the fluorescent labelled *E. coli *test sample are listed in the Table 1, and compared to the manual annotation. The results in Tables 1 and 2 reveal the high segmentation accuracy and generality of the method. Illustrative examples from test samples are presented in Figure 2.

**Table 1:** Test results on confocal images of *E. coli *cells expressing a fluorescent protein, MS2d-GFP

Test Case	No. images	No. cells	TP	Over -seg. (total/ %)	Under -seg. (total/ %)	FN (total/ %)	Segmentation accuracy (%)
Dense	10	7947	7335	236/2.96	170/2.13	206/2.59	92.30

Medium	10	4014	3616	87/2.16	184/4.58	127/3.16	90.10

Sparse	10	857	817	20/2.33	16/1.86	4/0.46	95.33

Total	30	12818	11768	343/2.67	370/2.88	337/2.63	91.80

TP and FN stand for true positive and false negative, respectively.

**Table 2:** Test results from phase contrast images of *E. coli *cells, from fluorescence images of *S. aureus*, and from epifluorescence images of *E. coli*.

Test Case	No. images	No. cells	TP	Over seg. (total/ %)	Under-seg. (total/ %)	FN	**Seg**. accuracy (%)
Phase contrast - *E. coli *	4	381	376	3/0.787	2/0.52	0/0	98.69

*S. aureus*	3	768	710	18/2.34	40/5.21	0/0	92.45

Epi - *E. coli*	3	160	146	5/3.12	6/3.75	3/1.87	91.25

Total	10	1309	1232	26/1.99	48/3.67	0.23	94.12

TP and FN stand for true positive and false negative, respectively.

**Figure 3:**
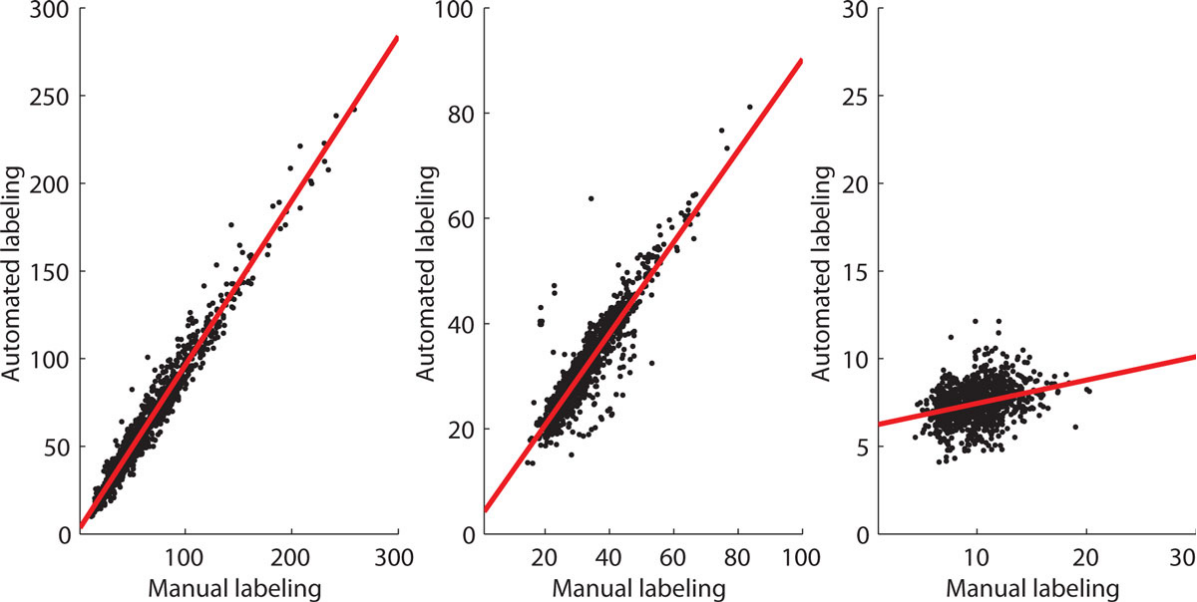
**Scatter plot of total cell intensity (left plot), length of the cell (middle plot) and width of the cell (right plot) in pixel by pixel comparison test**.

As a proof of concept, the efficiency of the segmentation method is evaluated against manually labelled cells at pixel level. This is carried out for three illustrative features: total cell intensity, cell length, and cell width. The test comprises approximately 1100 GFP labelled *E. coli *cells collected from 13 images. Figure 3 shows the quantitative results in scatter plots with a least square regression line. The horizontal axis represents the results from manual labelling and the vertical axis represents the results from the automated segmentation. A strong correspondence between manual and automated segmentation is evident. The correlation coefficients for the listed features were 0.98 (total cell intensity), 0.91 (cell length) and 0.31 (cell width), respectively. The correlation of the cell width feature is lower due to substantial inaccuracy in the manual segmentation of this feature. The presence of cells dividing was the other main cause for this error rate.

**Figure 4:**
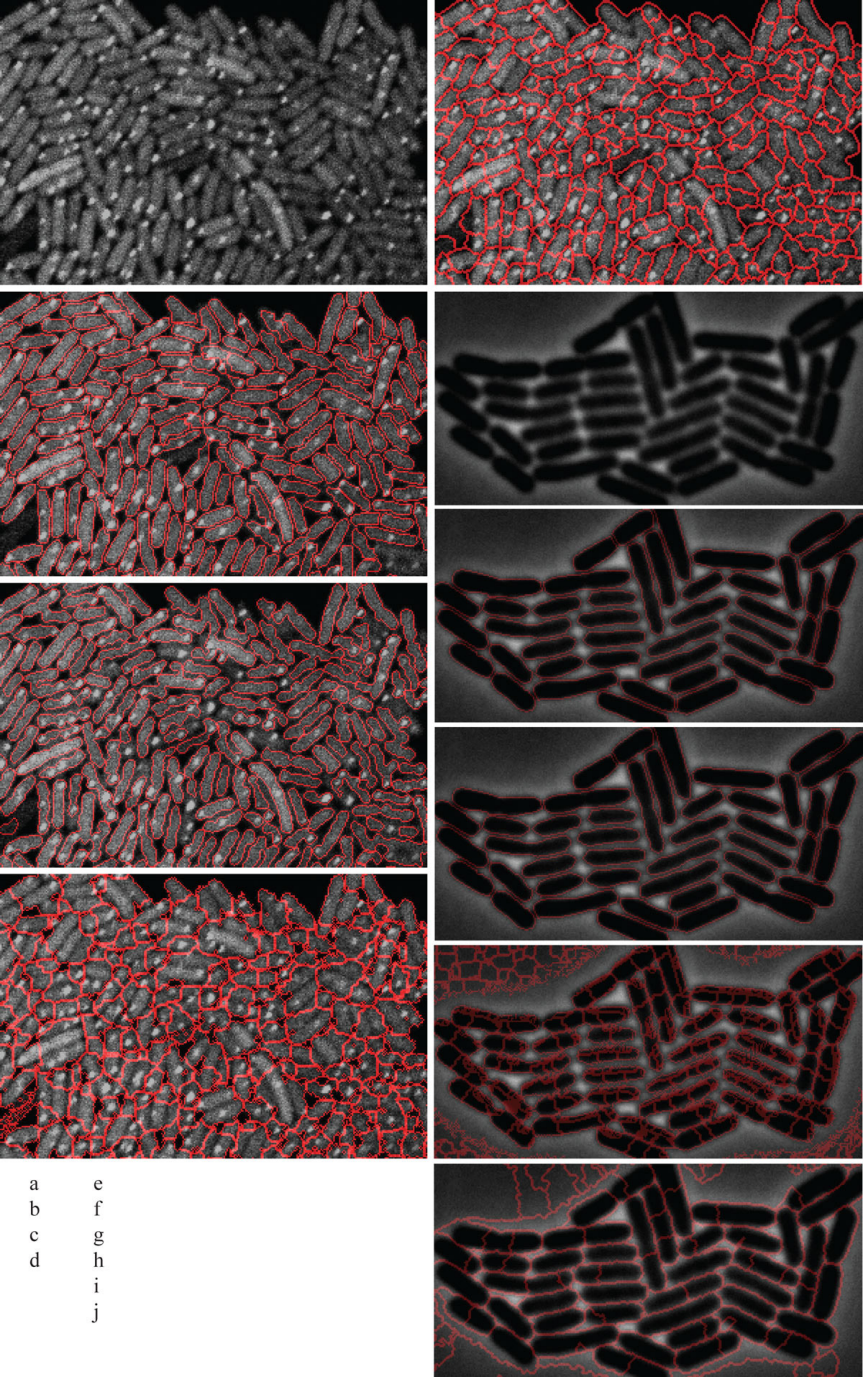
**Segmentation comparison result**. Fluorescent protein labelled *E. coli *cells captured with confocal microscope, (b) Segmented result of (a) by the proposed method, (c) Segmented result of (a) by Schnitzcells software, (d) Segmented result of (a) by the Farsight toolkit, (e) Segmented result of (a) by Cellprofiler, (f) *E. coli *cells captured with phase contrast microscope(Source [9]), (g) Segmented result of (f) by the proposed method, (h) Segmented result of (f) by Schnitzcells software, (i) Segmented result of (f) by the Farsight toolkit, (j) Segmented result of (f) by Cellprofiler. Figures are labelled as follows: on the left side, from top to bottom, are figures a) to d). On the right side, from top to bottom, are figures e) to j). This labelling is also indicated in the bottom left of the image.

**Figure 5:**
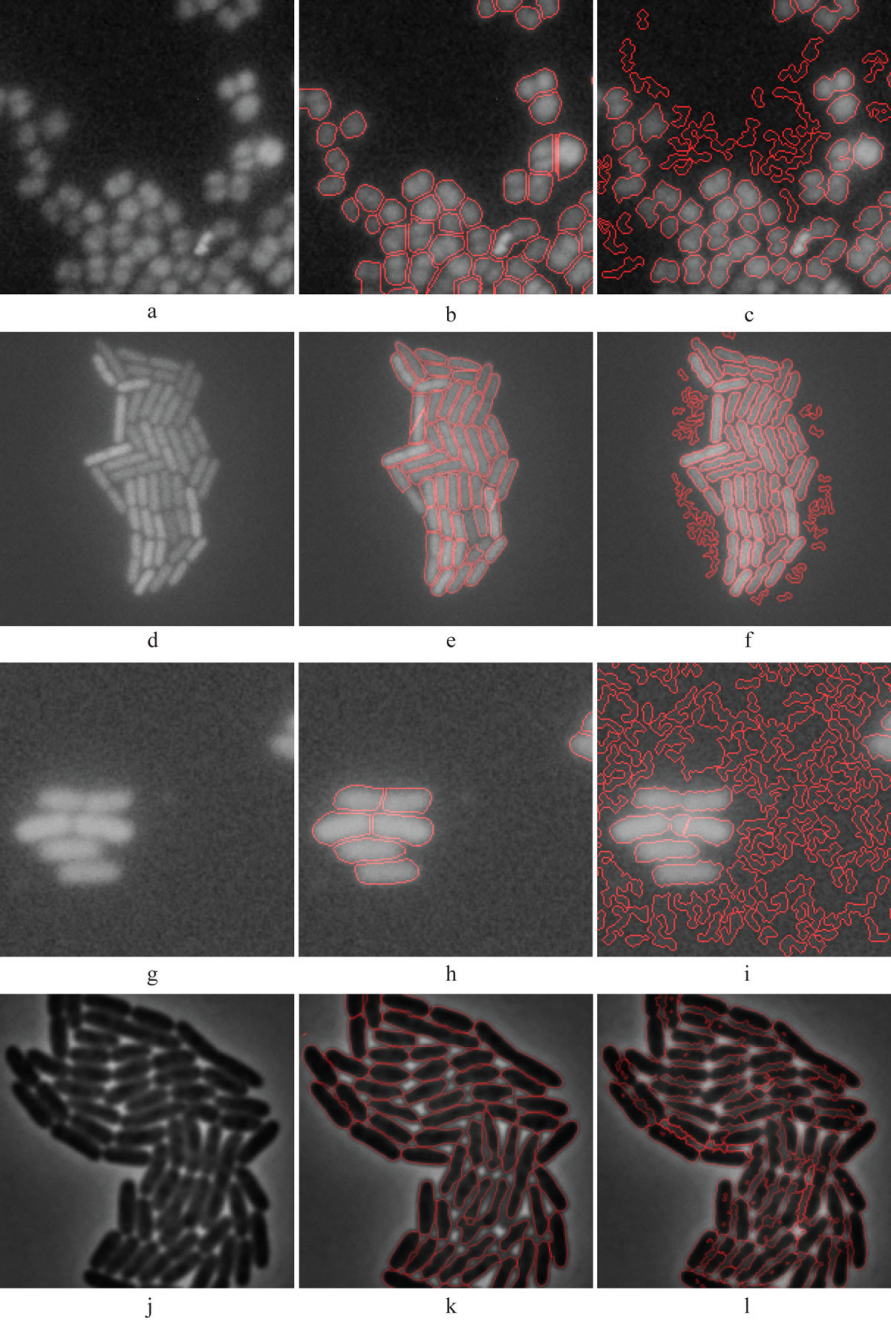
**Comparison between the proposed method and Schnitzcells software**. (a) Fluorescent protein labelled *Staphylococcus *cells in Epifluorescence microscopy image, (b) Segmented result of (a) by the proposed method, (c) Segmented result of (a) by Schnitzcells software, (d) Fluorescent protein labelled *E. coli *cells captured with confocal microscope (Source [9]), (e) Segmented result of (d) by the proposed method, (f) Segmented result of (d) by Schnitzcells software, (g)) Fluorescent protein labelled *E. coli *cells captured with Epifluorescence microscope, (h) Segmented result of (g) by the proposed method, (i) Segmented result of (g) by Schnitzcells software, (j) *E. coli *cells captured with phase contrast microscope (Source [12]), (k) Segmented result of (j) by the proposed method, (l) Segmented result of (j) by Schnitzcells software.

The cell segmentation accuracy of the algorithm is next compared with three state-of-the-art cell image analysis platforms, namely, Cellprofiler [8], Farsight [10], and Schnitzcells [9]. For the comparison to be unbiased, test samples were included from publicly available online repositories [8-10]. A set of sample results is shown in Figure 4. In general, we found the method proposed here to outperform the others in segmentation accuracy. Schnitzcells was the second best in *E. coli *segmentation (Figure 5). To further compare the proposed method and Schnitzcells we extended the evaluation. This additional test is carried out using publicly available bench mark images for cell counting [8]. The benchmark data contains roughly 2162 human HT29 colon cancer cells in 6 images. The cells were manually labelled and scored by two human observers and the average of the manual score is considered the ground truth. The human labelling had a mean absolute deviation of 11% and the best know result for this data set was attained by Cellprofiler, with a mean absolute deviation of only 6.2% [8]. In this benchmark data, our method exhibited a mean absolute deviation of only 1.79%.

Finally, we consider the usability of the novel method. In addition to accurate segmentation, the number of parameters and a robust performance in the parametric space are critical aspects of an accurate segmentation method. Ideally, an automated method should have as few free parameters as possible, and their tuning should be intuitive. Also, the optimal range of parameters should be large enough for tuning properly. Such 'parametric robustness' is what enables the segmentation method to be applicable to large scale analysis without the need for significant effort regarding the parameter tuning.

The proposed method has only 4 free parameters for tuning, namely, maximum cell size, scaling or resolution level, decomposition level and threshold window size, which affect the segmentation accuracy. The first three are intuitive in the sense that the maximum cell size can be estimated from knowledge of the phenotype of the cells (or a quick observation of the test samples) and microscope settings. The maximum scaling factor should be smaller than the cell width and, finally, the decomposition level should be less than the maximum scaling factor.

**Figure 6:**
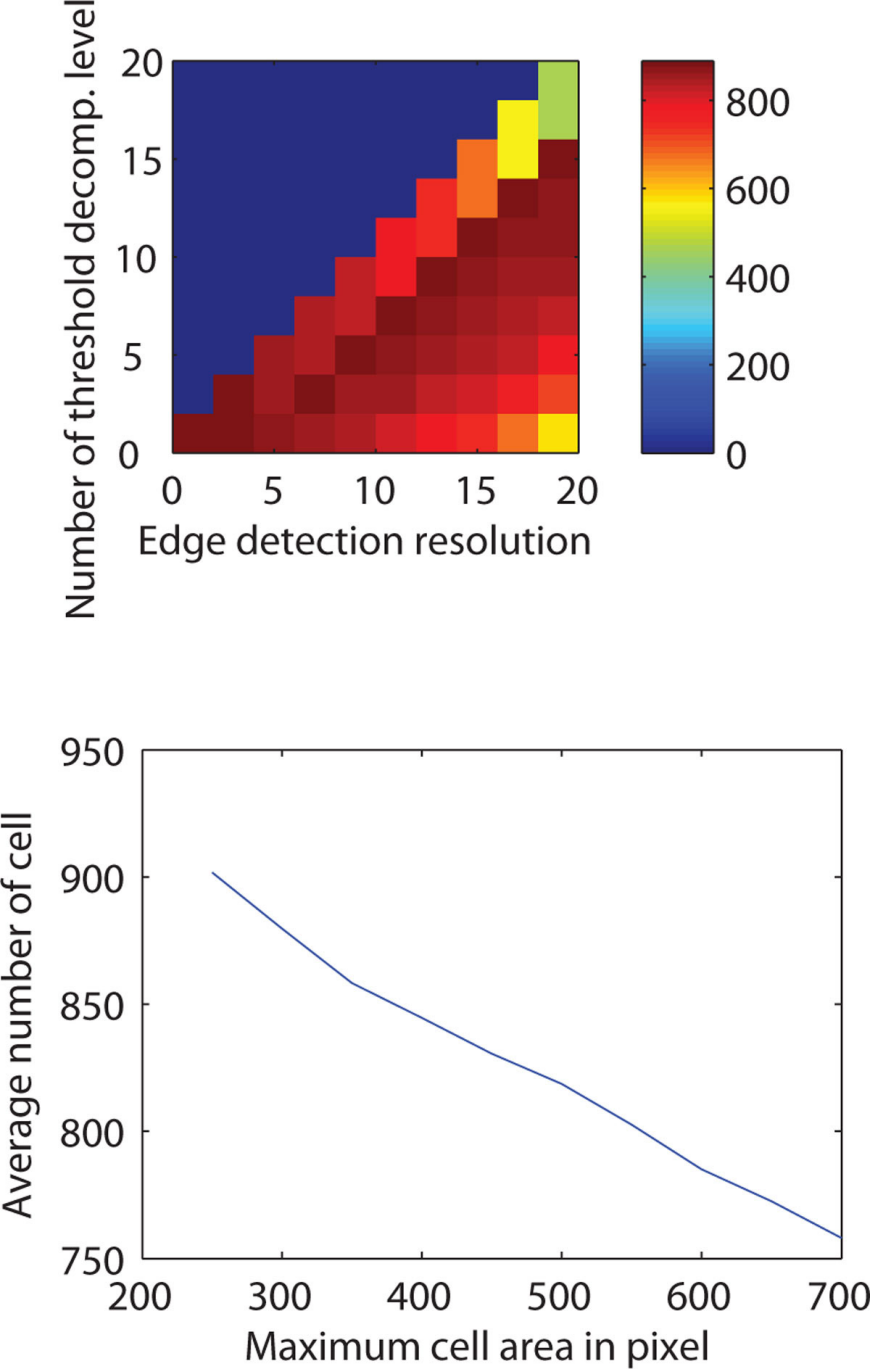
**Parametric robustness**. Top: Number of detected cells (color encoded, encoding scheme is shown on right side color bar) for different combinations of edge detection resolution level (X-axis) and number of maximum threshold decomposition level (Y-axis). Bottom: Number of cells detected for different values of the free parameter 'length of the cell'.

The parametric robustness of MAMLE is studied in 10 sample images containing approximately 8000 cells. It considers cell count as an objective metric for evaluation. The result is shown in Figure 6. Figure 6 (top) illustrates the effect of varying the scale level and the decomposition level. The cell count is stable across a wide range of the respective parameters. The results in the Figure 6 (bottom) are obtained by varying the maximum cell size. They indicate that this parameter affects the cell count in a linear fashion. Note that the coefficient of variation of cell count was much lower (0.057) than the coefficient of variation of the input parameter 'maximum cell size' (0.32). Thus, it can be stated that the algorithm obtains robust cell detection results within a wide range of numerical settings of the free parameters. The parameter, 'threshold window size' depends on the spatial distribution of cell background and foreground illumination levels. The largest possible window with homogeneous illumination level is the optimum for this parameter.

## Conclusions and discussion

Automated cell segmentation with high accuracy is a major challenge as well as a necessity towards high throughput analysis in cell biology, whose research is increasingly relying on *in vivo *single-cell studies. Here, we presented a novel method for automatically segmenting cells within colonies from microscopy images. The segmentation scheme exploits image de-noising techniques in transform-domain followed by multi-resolution edge detection and threshold decomposition for generating initial segmentation results. Then, a machine learning procedure is carried out to learn morphological shape parameters from the initial segmentation. Next, a likelihood optimization based splitting and maximum likelihood estimate based merging steps are executed to construct the accurate segmentation result.

The method was primarily evaluated for segmenting GFP labelled *E. coli *cells, but it was also tested for different cell types and imaging modalities. The test set comprises both de novo data set as well as samples from publicly available off-the-shelf benchmark data set. The segmentation results were found highly accurate by manual inspection, and denote high segmentation accuracy when compared with existing methods. The main strength of MAMLE relies on its ability to segment dense cell colonies as well as it robustness across a wide range of imaging modalities of different cell types.

Relevantly, the parameter selection is limited to three parameters, whose setting is intuitive. Either knowledge of the cells' morphology or a quick observation of the images, along with knowledge on the magnification settings of the microscope suffice to introduce parameter values that lead to robust results. Nevertheless, the overall performance was found robust to sub-optimal parameter settings as well. A forth parameter, 'threshold window size', as discussed, should be obtained from the spatial distribution of cell background and foreground illumination levels.

In the future, MAMLE can be extended in several ways. One plausible improvement is to add the possibility of training the method beforehand and update the trained knowledge base at runtime, rather than building the entire knowledgebase at runtime. This may be of use to research groups that focus on a specific organism and desire close to optimal results without the need to test the method and its parameters for each study.

## List of abbreviations used

BM3D: Block-Matching and 3D filtering; FN: False Negative; GFP: Green fluorescent protein; MAMLE: Multi-resolution Analysis and Maximum Likelihood Estimation; NP-hard: Non-deterministic Polynomial-time hard; TP: True positive.

## Competing interests

The authors declare that they have no competing interests.

## Authors' contributions

SC has planned the study, developed necessary software and executed the testing for result preparation. MSK has cultured the samples cells, provided image data for evaluation empirical evaluation and evaluated the result. ASR conceived the study. ASR and SC wrote the manuscript. All authors performed research.

## Availability and additional materials

http://www.cs.tut.fi/~sanchesr/CellSegment/index.htm

## Declarations

The publication costs for this article were funded by the Finnish Funding Agency for Technology and Innovation (grant no. 40226/12).

This article has been published as part of BMC Bioinformatics Volume 14 Supplement 10, 2013: Selected articles from the 10th International Workshop on Computational Systems Biology (WCSB) 2013: Bioinformatics. The full contents of the supplement are available online at http://www.biomedcentral.com/bmcbioinformatics/supplements/14/S10.
